# Quantifying geographical accessibility to cancer clinical trials in different income landscapes

**DOI:** 10.1016/j.esmoop.2022.100515

**Published:** 2022-06-21

**Authors:** G. Tini, D. Trapani, B.A. Duso, P. Beria, G. Curigliano, P.G. Pelicci, L. Mazzarella

**Affiliations:** 1 Department of Experimental Oncology, IEO European Institute of Oncology IRCCS, Milano, Italy; 2 Division of Early Drug Development, IEO European Institute of Oncology, IRCCS, Milano, Italy; 3 Department of Architecture and Urban Studies (DAStU), Politecnico of Milano, Milano, Italy; 4 Department of Oncology and Hemato-Oncology, University of Milano, Milano, Italy

**Keywords:** accessibility, geography, oncology, clinical trials, optimization, inequalities

## Abstract

**Background:**

Clinical trials are increasingly perceived as a therapeutic opportunity for cancer patients. Favoring their concentration in few high-expertise academic centers maximizes quality of data collection but poses an issue of access equality. Analytical tools to quantify trial accessibility are needed to rationalize resources.

**Materials and methods:**

We constructed a distance-based accessibility index (dAI) using publicly available data on demographics, cancer incidence and trials. Multiple strategies were applied to mitigate or quantify clear sources of bias: reporting biases by text mining multiple registries; reliability of simple geographical distance by comparison with high-quality travel cost data for Italy; index inflation due to highly heterogeneous cancer incidence by log-transformation. We studied inequalities by Gini index and time trend significance by Mann–Kendall test. We simulated different resource allocation models in representative countries and identified locations where new studies would maximally improve the national index.

**Results:**

The dAI approximated well a more realistic but not widely applicable travel cost-based index. Accessibility was unevenly distributed across and within countries (Gini index ∼0.75), with maximal inequalities in high- and upper-middle-income countries (China, United States, Russian Federation). Over time, accessibility increased but less than the total number of trials, most evidently in upper-middle-income countries. Simulations in representative countries (Italy and Serbia) identified ideal locations able to maximally raise the national index.

**Conclusions:**

Access to clinical trials is highly uneven across and within countries and is not mitigated by simple increase in the number of trials; a rational algorithmic approach can be used to mitigate inequalities.

## Highlights

• Accessibility to cancer clinical trials grew less than total number of trials over time in upper-middle-income countries.
• Accessibility is unevenly distributed, with maximal inequalities in high- and upper-middle-income countries.
• Simulation of resource allocation can identify ideal locations able to raise the national accessibility index.

## Introduction

Clinical trials are essential for cancer treatment, to generate new therapeutic paradigms and optimize the quality of care. The availability of clinical trials expands therapeutic options for patients, who may derive tangible benefits from access to innovative treatments.^1^^,^^2^ However, the conduct of clinical trials implies substantial structural and organizational investments that can be optimally managed by concentrating resources in academic medical centers,^3^ resulting often in heterogeneous trial availability based on the geographical distribution/localization of the investigation sites, impeding patients’ access on a large scale. Accessibility to health care involves spatial (e.g. geographical) and non-spatial (e.g. social, financial and psychological) aspects, including service availability and affordability, out-of-pocket costs, travel distance and personal satisfaction, which can all become critical barriers to the use of health care services.^4^^,^^5^ These considerations become even more relevant for cancer patients who require repeated visits for diagnosis and treatment^6^ and are often in high psychological distress. Transportation is one of the main challenges in patients’ access to clinical trials^7^: long time travels (estimated as >30-35-min travels in a study in Washington^8^) may dissuade participation, while a proper study site selection with a large nearby population pool is correlated to larger recruitment.^9^ Previous research, mostly conducted in the United States, showed great differences in clinical trial access between coastal versus internal states^10^^,^^11^ and rural versus urban areas, which also correlated with survival outcomes.^12^^,^^13^ At the global level, similar studies revealed a migration of clinical research toward countries with transitional economies such as China or Brazil, thus increasing the possibility for people once excluded from innovative treatments to obtain access.^14^^,^^15^

The geography of cancer clinical research has been investigated also in other countries, including China,^16^ Eastern Europe, the Russian Federation^17^ and Nigeria.^18^

Here, based on publicly available information, we propose an index to measure geographical accessibility to interventional cancer clinical trials on a global scale. Our index considers the available number of trials (supply), cancer patients (demand) and distance from the nearest investigation site and is derived from the gravity model proposed by Hansen in his seminal work in 1959,^19^^,^^20^ which has been largely used to assess accessibility to health care services.^13^ We did not consider the local competition proposed in Hansen’s model since typically the competitive dynamics among potential candidates for a given trial take place at the global scale and not at the local scale, with patients potentially eligible for more than one clinical trial.^21^

We mapped trends in accessibility in space and time and investigated the correlation with national income to study the global impact of cancer clinical research in different landscapes. Finally, through model simulations, we defined an algorithm to identify optimal geographical locations in which an increase in trial output would maximize national accessibility.

## Materials and methods

Extensive methods are presented in the Supplementary Methods, available at https://doi.org/10.1016/j.esmoop.2022.100515. Briefly, to calculate our distance-based accessibility index (dAI), we obtained:

- clinical trial data (indication, sponsor, trial locations) from ClinicalTrials.gov (CTG);
- worldwide geospatial and population data from the Gridded Population of the World v4,^22^ which maps the world on a uniform grid with points every 30 arc-min (∼55 km) on latitude and longitude directions; and
- national cancer incidence rates per 100 000 cases from GLOBOCAN 2018.^23^

We then calculated a dAI for each population grid point *i* as the number of trials *n*_*j*_ activated in the closest trial center *j* divided by the square root of the distance *d_ij_* from the grid point to the center (shown to best describe the behavior of human mobility when traveling at the average scale distance between patients and clinical sites,^24^^,^^25^ Supplementary Table S1, available at https://doi.org/10.1016/j.esmoop.2022.100515) and the logarithm of the estimated number of patients *s*_*i*_ in the grid point *i*, according to the formula:

$$dAI\left(i\right)=\frac{n_{j}\;}{\log\;s_{i}}\left(\frac{1}{\sqrt{d_{ij}}}\right),\;i=1\ldots N_{C}$$

*N*_*C*_ is the total number of grid points for the considered nation, while the estimated number of patients is defined as *s*_*i*_ *= p*_*i*_ *× r*_*c*_, that is the product of the population density at the grid point *i* (*p*_*i*_) and the national cancer incidence (*r*_*c*_). For each country, the national accessibility index is the average of the local accessibilities *dAI(i)* weighted on the logarithm of total population with cancer. Bias mitigation and control strategies and the maximization algorithm are described in the Supplementary Methods, available at https://doi.org/10.1016/j.esmoop.2022.100515.

## Results

### Dataset description

**Figure 1:**
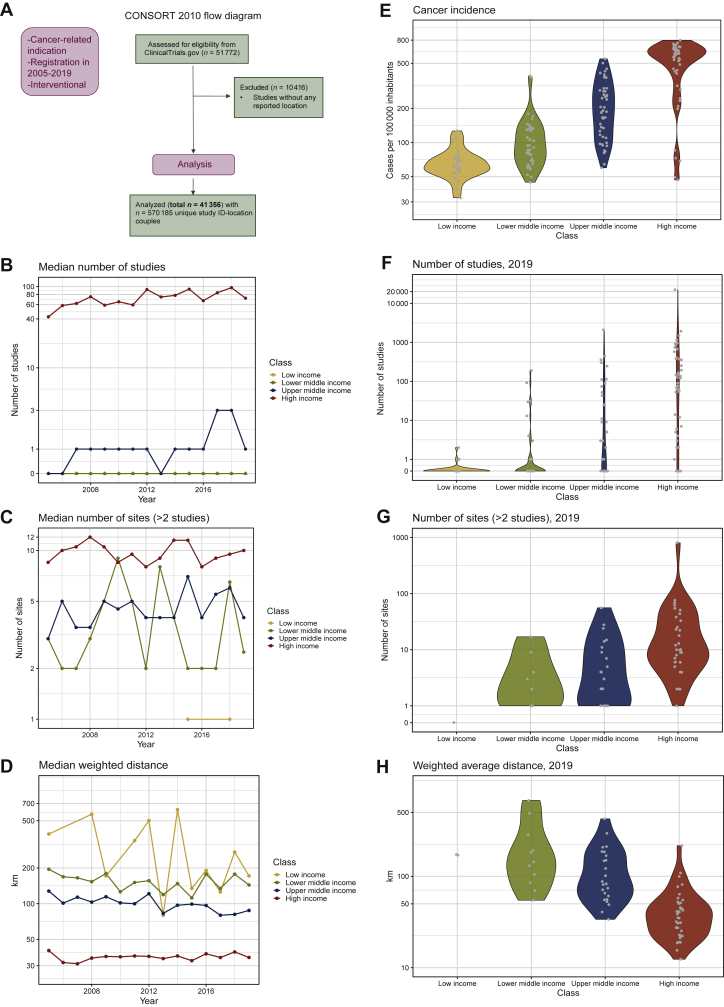
**Overview of variables in the four income classes.** (A) Consolidated Standards of Reporting Trials (CONSORT) 2010 flow diagram. (B) Trend of the median number of cancer clinical trials registered from 2005 to 2019. (C) Trend of the median number of sites with at least three cancer studies from 2005 to 2019. (D) Trend of the average distance to the closest site with cancer clinical trials in the period 2005-2019. (E) Violin plot representing distribution of 2018 cancer incidence. (F) Violin plot showing the distribution of the number of cancer clinical trials in 2019 for the different income classes. Grey dots represent different countries. (G) Violin plot with distribution of the number of sites with at least three cancer studies in 2019 for the different income classes. Grey dots represent different countries. (H) Violin plot representing distribution of distance, weighted on population, in 2019 for the four income groups. Grey dots represent different countries.

Our analyses are based on a dataset of cancer interventional clinical trials with available city-level geographical location. The dataset was necessarily centered around CTG, the only registry that collects detailed geographical information, which is also naturally biased toward North American trials. The CTG dataset, used for all subsequent analyses, includes 51 772 trials registered between 2005 and 2019 (Figure 1A), for a total of 570 185 study–location associations. City-level geographical information is not available in other databases and in particular in the comprehensive World Health Organization (WHO) database that includes most non-American national or continental registries (e.g. EudraCT, see Supplementary Table S2, available at https://doi.org/10.1016/j.esmoop.2022.100515, for the complete list). Data loss due to representation bias was quantified through text mining (Supplementary Data, available at https://doi.org/10.1016/j.esmoop.2022.100515) and showed no substantial information addition (defined as a difference of >25% between the two registries, see Supplementary Table S3 and Figure S1, available at https://doi.org/10.1016/j.esmoop.2022.100515) from WHO registry for most countries (88.6%). Countries with substantial differences were enriched in the lower-middle-income group and include countries of known geopolitical instability or isolation (Iran, Syria), though with some notable exceptions (China, Japan). For low-income countries differences were not substantial, given the overall low number of active trials (Supplementary Figure S2, available at https://doi.org/10.1016/j.esmoop.2022.100515). These differences remained stable over time, highlighting that sub-representation in CTG is systematic and constant for specific nations: analyses for these countries should be considered less reliable and are flagged in the study.

### Overview of the cancer clinical trial landscape

Despite a general increase (61.5%) in the number of trials from 2005 to 2019 (Supplementary Table S4, available at https://doi.org/10.1016/j.esmoop.2022.100515), 60/175 countries (34.3%, of which two-thirds belong to the low- and lower-middle-income groups) remained with no access. Among the other countries, only 56 (33.1% of the total) had new trials registered every year (Supplementary Table S5, available at https://doi.org/10.1016/j.esmoop.2022.100515).

In 2019, public-funded studies were more numerous than industry-funded studies (2981 versus 951) and growing more with respect to 2005 (76.5% versus 48.6%). Most of the countries that showed strong increase are in the upper-middle-income group (e.g. China, Colombia; Supplementary Figure S3, available at https://doi.org/10.1016/j.esmoop.2022.100515).

Industry-sponsored trials showed a significant bias for phase I trials in 2019 (39.5% of total studies funded by industry, chi-square test *P* value <2.2 × 10^−16^), which, in their early phase, also showed the strongest increase over time (1700% since 2005). Publicly funded trials are instead enriched in phase II (31.7%, chi-square *P* value <2.2 × 10^−16^).

### Trial accessibility by income groups and countries

To quantify intra- and inter-country inequalities in access to trials, we developed a dAI. The adequacy of the dAI in summarizing trial accessibility was confirmed by a comparison with travel cost-based indices (tAI) computed for Italy, for which high-quality travel cost data are available^24^ (Supplementary Figures S4-S6, available at https://doi.org/10.1016/j.esmoop.2022.100515). The dAI was systematically lower but well correlated both across locations in 2019 (Pearson’s correlation ≥0.71) and at the national level over time (Pearson’s correlation ≥0.96).

**Figure 2:**
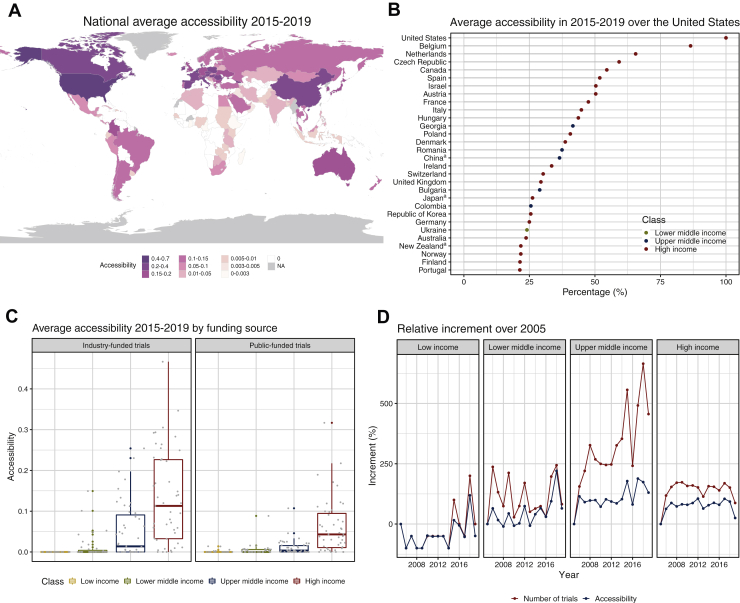
**Average national accessibility and comparison with trial number.** (A) Average national accessibility in the period 2015-2019. Countries with not available data are colored in grey, and those with no accessibility to clinical trials in white. (B) Comparison of the average national accessibilities with that of the United States in the period 2015-2019. Percentage of USA accessibility reached by the top 30 world countries is displayed. ^a^Countries with significant difference from trials in World Health Organization (WHO) registry. (C) Comparison in average national accessibility to differently funded trials: industry (left panel) and public (right panel) in the period 2015-2019. Boxplots describe distribution of accessibility in different income classes, represented by different colors as in B. (D) Comparison in average relative increments over 2005 for accessibility and number of trials registered. Trends from 2005 to 2019 are displayed for each income class.

Index parameters (cancer incidence, number of trials per location, number of trial locations, distance from the nearest trial location) were retrieved completely for 162/175 countries (47 high-, 47 upper-middle-, 40 lower-middle- and 28 low-income countries). Expectedly, the median number of trials and sites was positively correlated with nation income status, whereas average distance was negatively correlated (Figure 1B-D). In 2019, all variables exhibited large within-group variation (Figure 1E-H), which is reflected in the heterogeneous distribution of average national accessibility in the period 2015-2019 (Figure 2A, complete data and Supplementary Table S6, available at https://github.com/translational-oncology-lab/CTAccessibilityTool). Since the accessibility index for the United States remained relatively stable over time (Supplementary Figure S7, available at https://doi.org/10.1016/j.esmoop.2022.100515), we expressed every other nation’s accessibility relative to the United States. Expectedly, the majority of countries with high accessibility belong to the high-income group. Some exceptions (Georgia, Romania, China, Bulgaria, Colombia and Ukraine) are found in the upper- and lower-middle-income groups (Figure 2B); of note, these countries have cancer incidence higher than their income group average (Supplementary Table S6 available at https://github.com/translational-oncology-lab/CTAccessibilityTool). Accessibility to industry-funded clinical trials was found to be generally higher than accessibility to public-funded trials (Figure 2C) and, similarly to accessibility to phase III trials, large in high- and upper-middle-income countries (Supplementary Figure S8, available at https://doi.org/10.1016/j.esmoop.2022.100515). Accessibility to clinical trials in poorer countries such as African and the Middle East ones is mainly due to the presence of public-funded trials.

Accessibility was correlated with total registered trials over time, but accessibility grew less rapidly than trial numbers in all income classes (Figure 2D), most strikingly for the upper-middle-income group, as highlighted by analyzing the area under the curve (AUC) of time versus increase (Table 1): accessibility-AUC accounts for just 33.8% of trials-AUC for the upper-middle-income class (accessibility-AUC/trials-AUC), and then increases to 49.3% for the lower-middle-, 54.5% for the high- and 79.9% for the low-income classes.

**Table 1:** AUC for relative increments over 2005 in trial number and accessibility, and correlation between increments in the four income classes

Income class	AUC trials	AUC accessibility	Pearson’s correlation
Low	1000.0	799.9	0.97 (*P* value: 4.0e−9)
Lower middle	1630.4	803.8	0.71 (*P* value: 3.2e−3)
Upper middle	4575.6	1546.2	0.89 (*P* value: 1.1e−5)
High	2014.9	1098.0	0.91 (*P* value: 3.2e−6)

AUC, area under the curve.

**Figure 3:**
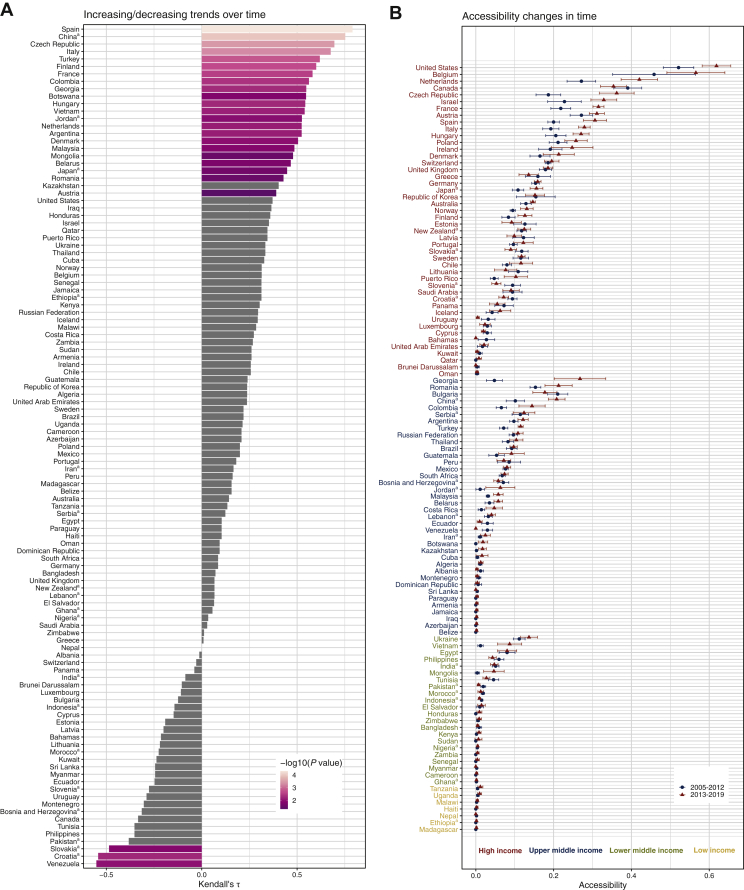
**National accessibility changes in time.** (A) Countries with increasing (Kendall’s τ statistics >0) and decreasing (Kendall’s τ statistics <0) accessibility trends in 2005-2019. Only countries with mean values in period 2005-2012 and 2013-2019 both different from 0 are displayed. Bars are colored according to the significance of the *P* value from the Mann–Kendall test. Countries with not significant trends (*P* > 0.05) are colored in grey. ^a^Countries with significant addition of World Health Organization (WHO) trials from sensitivity analysis. (B) Changes in the national accessibility between period 2005-2012 and 2013-2019. Dots represent the mean value in the corresponding time range, and segments the standard deviation. For each income class, countries with both mean value different from 0 are displayed. ^a^Countries with significant difference from trials in WHO registry.

Time trend analysis identified 25 countries with significant upward or downward trends (positive or negative Kendall’s τ, Figure 3A and B, Supplementary Table S7, available at https://github.com/translational-oncology-lab/CTAccessibilityTool), with Spain showing the strongest increase (53.2%, Kendall’s τ 0.79, *P* value 5.0 × 10^−5^). Countries with largest relative increases between the time periods 2005-2012 and 2013-2019 in the upper- and lower-middle-income groups showed wide fluctuations (Supplementary Table S7, available at https://github.com/translational-oncology-lab/CTAccessibilityTool). No significant trend was identified for low-income countries.

To measure the reliability of the dAI, we compared it with travel cost-based indices (tAI) in Italy, for which travel cost between locations has been carefully computed with high spatial resolution.^24^ Comparing dAI versus four different tAIs for 2019 showed that dAI is in general slightly higher and led to a lower Gini index (Supplementary Figures S4 and S5, available at https://doi.org/10.1016/j.esmoop.2022.100515), suggesting that distance may ‘underestimate’ the difficulties in reaching a specific location. Reassuringly, correlation between dAI and tAIs is high, both across locations in 2019 and at the national level over time (Supplementary Figures S5 and S6, available at https://doi.org/10.1016/j.esmoop.2022.100515). Thus, despite differences in absolute numbers, distance alone acceptably approximates relative differences in accessibility and appears sufficiently informative, especially in nations in the high- and upper-middle-income groups, comparable to Italy in terms of infrastructures.

### Disparities in accessibility distribution

**Figure 4:**
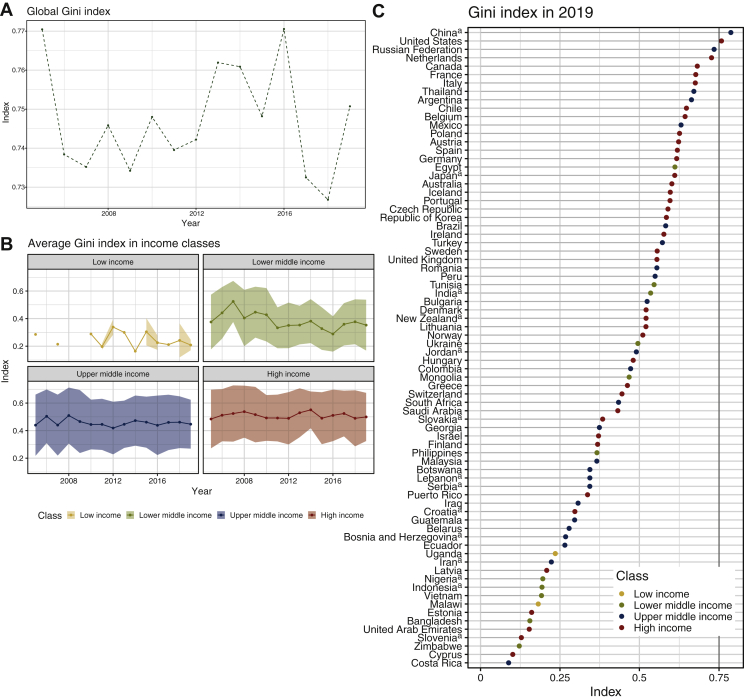
**Inequalities in accessibility distribution.** (A) Global inequality trend from 2005 to 2019. The black dotted line represents the global Gini index level of inequality. (B) Inequality trends from 2005 to 2019 in different income classes. Gini index for low income in 2006, 2008 and 2009 is null due to lack of clinical studies. (C) Within-country inequalities in the distribution of accessibility in 2019. Only countries with at least one cancer trial in 2019 are displayed. The vertical black line indicates the global Gini index in 2019. ^a^Countries with significant difference from trials in World Health Organization (WHO) registry.

Both global and internal inequalities, measured using the Gini index, are generally high: maximum was reached at 0.79 in 2016 (Figure 4A). The countries with wider internal heterogeneities in 2019 belonged to the high- and upper-middle-income groups (Figure 4B), with an average Gini index above 0.5 and 0.45, respectively. In 2019 (global Gini index = 0.78), a total of 37/162 countries showed an index larger than 0.5 (Figure 4C): China was identified as the country with the highest Gini index, equal to 0.79 followed by the United States (Gini index = 0.76); those countries have poles of urbanization hosting the majority of clinical trials.^11^^,^^16^ Removing from the analysis, ‘flagged’ countries with substantial CTG underrepresentation did not have any major impact on global and by-income-class Gini index, which only slightly augmented (Supplementary Figure S9, available at https://doi.org/10.1016/j.esmoop.2022.100515, ranges for global Gini index: 0.74-0.78 after removal versus 0.73-0.77 before removal).

### An algorithm to inform health resource allocation

We explored whether our accessibility index can inform resource allocation models that may maximize geographical accessibility and enhance efficiency in health planning.

We first generated simulated scenarios to evaluate which variable would confer maximal gain (see Supplementary Material and Figure S10, available at https://doi.org/10.1016/j.esmoop.2022.100515). Expectedly, the largest gain was obtained by adding trial locations (Supplementary Figure S11, available at https://doi.org/10.1016/j.esmoop.2022.100515), as well as by decreasing population density (Supplementary Figure S12, available at https://doi.org/10.1016/j.esmoop.2022.100515), but significant shifts were also obtained by specific geographical configurations (Supplementary Figures S11 and S12, available at https://doi.org/10.1016/j.esmoop.2022.100515), suggesting that a careful choice of trial location (the variable that can be more easily conditioned by health policy measures) can have a major impact.

We then applied the simulation to real scenarios to identify the optimal geographical location for potential new trial sites with given size.

We selected countries that represent stable clinical research landscape in the income groups with more clinical trials: Serbia (upper-middle income) and Italy (high income). They have more studies and lower distance than their group median and also exhibit high cancer incidence (third interquartile) in their income group, respectively, of 547.4 and 691.2,^23^ indicative of a particular need for increasing accessibility in these specific countries.

**Figure 5:**
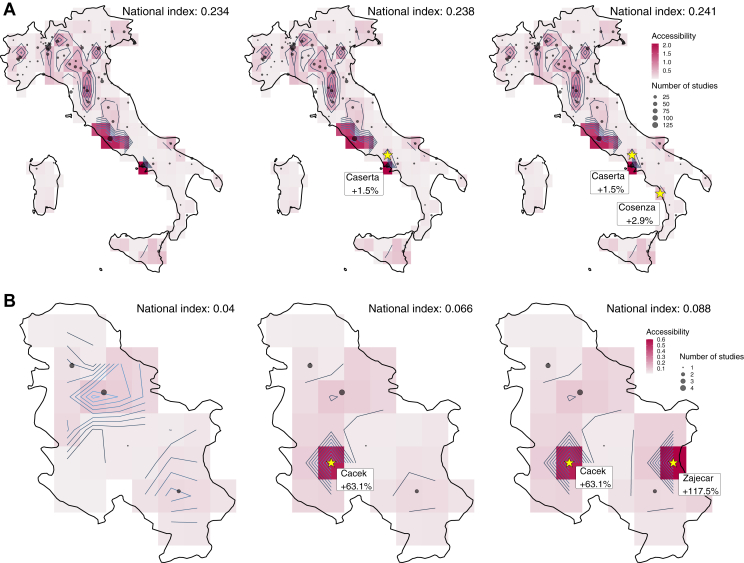
**Optimal locations and their impact on national accessibility.** (A) Local accessibility in Italy in 2019 for: the actual distribution of trials (left panel), the addition of studies in the first optimal location selected by our algorithm (middle panel) and the addition of trials in both optimal locations (right panel). Darker color shades represent larger local accessibility; study locations are displayed as points with increasing size for higher number of studies; stars represent optimal new locations selected. Lines with same accessibilities are displayed, together with national accessibility value, name of optimal locations and the relative increment obtained by their addition. (B) Local accessibility in Serbia in 2019 for: the actual distribution of trials (left panel), the addition of studies in the first optimal location selected by our algorithm (middle panel) and the addition of trials in both optimal locations (right panel). Colors, stars, dots and lines are the same as described in panel A.

Starting from the distribution of trials registered there in 2019, we identified optimal locations which improved national accessibility by 63.1% and 1.5%, respectively, for Serbia and Italy. With an additional location, the improvements are of 117.5% and 2.9% (Figure 5A and B).

## Discussion

Here we show that overall increases in trial numbers have not necessarily translated into improved accessibility, as trials continue to be run in recurrent locations. This is not unexpected given the significant resources required for running clinical trials,^3^ but as for other limited resources in the modern world, the quest for quality generates disparities in access that ultimately undermine impact^26^ and may put unmanageable pressure on site personnel.^27^ Our index provides a framework for quantitative analysis of the geographical constraints on patients’ recruitment, a research gap highlighted in previous analyses,^7^ and an algorithmic approach to inform policies for resource allocation.

Changes in the accessibility index intercepted significant shifts in national health care policies in emerging or rapidly growing economies (e.g. China and Colombia) or industry–academia partnership,^28^ further suggesting its usefulness as an indicator of health care output on the global scale.

Our results are in line with those of other studies that have highlighted major inequalities in trial access in specific diseases, such as studies on breast, lung and cervical cancers,^29^ or specific geographical contexts.^11^

We must stress that the accessibility index and the algorithm for optimal location identification are likely to be maximally informative for countries in the higher- and upper-middle-income groups, in which key parameters like cancer incidence or transport infrastructures are not subject to extreme variation, and in which access to basic cancer treatments has been achieved, such that access to clinical trials may represent a realistic opportunity for cancer treatment. These nations are also those in which our analysis identifies the most extreme variations in accessibility, either within nation (high income) or over time (upper-middle income), in line with their rapidly expanding economy.^14^ An appropriate case study is China: in 2018 most of the leading cancer trial units were concentrated in the eastern part of the country.^16^

Our index is less accurate and perhaps less useful in lower-middle- and low-income countries, in which trial reporting is likely much lower (most nations had not registered a single trial on CTG by 2019) and cancer incidence is significantly lower. We partially mitigated this factor by attenuating the importance of cancer incidence by log-transformation (a common and widely accepted procedure in composite global socioeconomic index construction^30^); furthermore, we measured the information loss associated with considering only trials registered in CTG, and we show that loss becomes significant mostly for nations of known geopolitical instability (e.g. Iran) for which often socioeconomic indices cannot be computed reliably. This residual bias risk is virtually impossible to mitigate as database structure and trial registering policies are heterogeneous.^31^ Albeit imperfect, the index might have intercepted initial health care policy shifts as in Mongolia, whose recent reforms drove a transition from a centralized to a more decentralized model, though not without its limitations,^32^^,^^33^ and Uganda, which keeps one of the few population-based registries in sub-Saharan Africa.^34^

The resource allocation algorithm unbiasedly identified areas that would maximize national trial accessibility. At least for Italy current data on trial distribution support the validity of our prediction, as the optimal sites are concentrated in Southern Italy in which high-volume academic sites are underrepresented compared to the northern area, relative to the size of the target population.^35^

The study has additional limitations. The most obvious is that our index does not take into account qualitative heterogeneities in cancer interventional trials (inclusion criteria, disease of study, type of treatment); patients in a particular geographical area may have more or less accessibility to a specific set of trials that may or may not be relevant for certain cancer settings. Although a more granular analysis with disease- or intervention-specific indexes is theoretically possible, in fact this is undermined by insufficient and heterogeneous reporting of trial parameters in trial repositories. This may be included in future editions of the index if controlled vocabularies and standard practices will be used in trial registering.^36^

To design a global index, we focused on widely available and standardized parameters. For instance, we used the number of clinical trials per location and not the planned number of patients. Although available as feature in the CTG registry, this information is highly fragmented. Similarly, we considered distance between locations, instead of travel time/cost, as these are only available for selected countries. The good correlation over time and across locations between distance-based and traveling cost-based indices supports the choice of distance on a wider scale, at least for nations with transport infrastructures not too different from Italy’s.

Finally, optimal locations predicted by our algorithm might not be necessarily ideal once all non-geographical factors are taken into account. These indications must be integrated in a broader perspective for national cancer research and control planning. This may be particularly relevant for low- and middle-income countries, where studies highlighted cultural and structural barriers beyond the political will,^37^ especially when framed in mixed models of funding (public and private efforts), that can affect adversely the intent to tackle public health problems^38^^,^^39^ and result in negligible population health impact. Therefore, the impact of clinical trial implementation on population health has been debated and an immediate positive impact should not necessarily be assumed. Positive outcomes of trial implementation have also been reported. For instance, it has been argued that health providers involved in clinical research deliver more consistent and evidence-based practice, possibly improving quality of care.^40^ An improved overall survival was associated with enrollment in clinical trials in several studies.^41^, ^42^, ^43^ Also, clinical trial centers can foster the connection of diverse elements of the health system, thereby serving as booster for more comprehensive health care delivery and enhancing scale-up, potentially resulting in better local access to cancer care.

The current model for clinical research leads to a punctuated distribution that creates high inequality in trial accessibility, leading to underrepresentation of communities of patients in rural or geographically isolated areas. The mathematical tools described in the present study may help to better quantitate such inequalities and facilitate the planning of trials that recruit patients from systematically neglected areas.^3^
