# Supplementary material for: Quantifying geographical accessibility to cancer clinical trials in different income landscapes
Source: ESMO Open. 2022 Jun 21;7(3):100515. doi: 10.1016/j.esmoop.2022.100515 (PMC9271515; doi:10.1016/j.esmoop.2022.100515)
Supplement: Supplementary material [file mmc1.docx]

**Supplementary material**

Supplementary methods

Supplementary results

Supplementary tables S1-S5

Supplementary figures S1-S12

**Supplementary Methods**

**Data sources and preprocessing**

We collected information on clinical trials from the online registry *clinicaltrials.gov* (<https://clinicaltrials.gov>) via the Clinical Trials Transformation Initiative database available at <http://www.ctti-clinicaltrials.org>, accessed on 29th July 2020.

We selected interventional clinical trials registered in the period 2005-2019 with cancer-related indication: cancer, tumor, tumour, sarcoma, blastoma, lymphoma, carcinoma, melanoma, neoplasm, mesothelioma, myeloma. We deliberately avoided considering 2020 in the time period of interest due to the well-known impact of the coronavirus disease 19 pandemics on clinical trials.^1,2^

Trials registered in more countries were counted as separate entities for each country. Studies without any reported site were excluded. Geographical locations of the hosting institutions were geolocated with R package tmaptools v.3 (OpenStreetMap Foundation, Cambridge, United Kingdom).

We integrated clinical trials data with geospatial and population data from the Gridded Population of the World v4^3^, obtained from [geoquery.org](http://geoquery.org), which maps the world on a uniform grid with points every 30 arc-minute (~55 km) on latitude and longitude directions. Population count in each grid point was provided for years 2005, 2010 and 2015. For in-between years, the population was assigned to the closest previous one (e.g. years from 2006 to 2009 assigned to 2005 population). Grid points with no population were excluded from the analysis, the remaining were assigned to their country by reverse-geolocation, for a total of 175 mapped countries.

Travelling cost data for different economical profiles of users (“economy” and “family”) in Italy were obtained from ref ^4^.

We obtained estimated national cancer incidence rates per 100,000 cases from GLOBOCAN 2018^5^ and grouped countries (“High”, “Upper-middle”, “Lower-middle” and “Low”, according to the World Bank (<https://data.worldbank.org/>) definitions.

**Accessibility index definition**

Given a country $C$ with $N_{C}$ points on the population grid and with cancer incidence rate $r_{C}$ (cancer cases per 100,000 inhabitants), we computed local accessibility for each point in the national grid $i, i=1\ldots N_{C}$. We estimated the number of cancer patients per year in the grid point $i$ as $s_{i}=p_{i}*r_{C}$, where $p_{i}$ is the total population in $i$; for each grid point we identified the closest location $j$ with at least one clinical trial. Distances were computed with the haversine formula (geosphere R package v. 1.5-10). Denoting by $d_{ij}$ the distance between$i$ and $j$, and by $n_{j}$the number of trials available in $j$, we defined the local accessibility index $dAI(i)$ as:

$$dAI\left( i \right)=\frac{n_{j}}{\log s_{i}}\left( \frac{1}{\sqrt{d_{ij}}} \right), i=1\ldots N_{C}$$

For each country, the national accessibility indexis the average of the local accessibilities $dAI(i)$ weighted on the logarithm of total population with cancer. The logarithmic function scales the impact of cancer incidence^6^ which is disproportionately low and potentially underestimated in Low and Lower-middle income countries.^7^ Points with $s_{i}\leq1$ were excluded from the national index computation, as they imply a negligible density of patients. We used the inverse power law of distance $d_{iJ}^{-\beta}$, $\beta=1/2$ as impedance function, previously shown to best describe the behaviour of human mobility when traveling at the average scale distance between patients and clinical sites^4,8^ (see Supplementary Table S1).

Besides the more general distance-based accessibility index dAI described above, we computed traveling cost-based accessibility index tAI for Italy, where traveling costs are available: we substituted the $d_{iJ}^{-\beta}$ factor with specific functions of traveling costs on street and local public transportation for different economical profiles of users^4^. Pearson’s coefficient was used to measure correlation between dAI and each tAI.

**Statistical analysis**

We checked the 2019 worldwide evolution of clinical trials number, phase and funding sources (industry, public agencies), by computing relative increments over 2005. Chi-squared test was used to quantify enrichment in trial phases.

We then restricted our analysis to countries with available income classification and incidence rate and computed local and national accessibility in the years 2005-2019. We analysed time trend changes in average accessibility and compared the average relative increments over 2005 of accessibility and registered trials: for this analysis we selected only countries with at least one study in 2005 (2 Low, 10 Lower-middle, 20 Upper-middle and 42 High income), then we compared the area under the curves (AUC) obtained with the computed trends in time. We also studied Pearson’s correlation between accessibility and trial number increments. With Mann-Kendall test we identified countries with a specific increasing/decreasing accessibility trend over time (positive/negative Tau and significance threshold at p-value<0.05) and computed statistics (mean, standard deviation) for national accessibility in the periods 2005-2012 and 2013-2019. To define most accessible countries, we divided national average accessibilities in the period 2015-2019 by USA value, the most stable over time. Lastly, we assessed inequalities in the distribution of accessibility globally and by income group with the Gini index, that ranges from 0 (homogeneous distribution) to 1 (total unequal distribution).

**Simulation of different resource allocation models**

To study the accessibility index in resource allocation model optimization, we first assessed index behaviour on synthetic configurations (Supplementary Figure S10).

Simulated configurations were designed starting from a 4 x 6 rectangular grid. An initial population of 100 was assigned to each of the 24 grid points. Five “research points” (A, B, C, D and E) were also located at the corners and in the center of the grid (not overlapping the grid points) and provided with five studies each. The research points were added to the grid in sequence to create different configurations (See Figure S10 for some examples). For each configuration we increased the number of studies to ten for all possible combinations of research points: for example, in configuration “ABE” we added five studies to the baseline for cases “A”, “B”, “E”, “A+B”, “A+E”, “B+E”, “A+B+E”. To consider more scenarios, for the configuration ABE we changed the population of two groups of grid points to 50 and 200 (Figure S10, panels C, D and E, F respectively). In this case we increased the number of studies of grid point both homogeneously (cases AB, AE, BE) and differently (cases 2AB, A2B, 2AE, A2E, 2BE, B2E). The notation “2xy” means that studies in x are always doubled with respect to y. Accessibility was computed for each scenario and results compared among configurations.

Then we implemented the model in countries chosen as representative of the most stable income groups, High and Upper-middle, with at least one trial open since 2015 and with high incidence rate: Serbia and Italy. For both countries we computed the annual median number of studies per location and their overall median, indicated by $m$. We took the 2019 location distribution as baseline and generated models by adding $m$ studies in each national grid point to the baseline. For each model we computed the national accessibility and selected as optimal location the grid point giving maximum accessibility. To identify a second location, we added the first location to the baseline and repeated the study addition step. We finally computed relative increments on 2019 baseline of the accessibility obtained with the two simulations.

All the analyses described have been performed using R version 3.6.2.

**Supplementary Results**

**Comparison between clinicaltrials.gov and other public registries**

To quantify how representative clinicaltrials.gov (CTG) is for non-American countries, we studied the intersection between CTG and i) EudraCT, the European registry for interventional clinical trials (<https://eudract.ema.europa.eu/>) in the period 2014-2019 and ii) the WHO registry in the period 2005-2019. To this aim we extracted EudraCT and WHO trials on cancer/tumor indication (R package ctrdata and through the International Clinical Trial Registry Platform [ICTRP] respectively).

***EudraCT analysis.*** We observed a total of 11,068 cancer trials (corresponding to 3,675 different EudraCT ID) registered on EudraCT in the period 2014-2019 and 70,464 European studies registered on CTG in the same period. To quantify the overlap of the two registries, we lowercased all information, removed punctuation and then compared EudraCT and CTG official titles, identification codes and acronyms. 1,723 EudraCT studies (2.4% of the European CTG studies) did not have a correspondence in CTG. Manual checking could further identify approximately 5% of the missing trials as overlapping, further suggesting that the fraction of European trials missing in our analysis is in fact negligible.

***WHO registry.*** We recovered 45,681interventional clinical trials from the WHO registry in the period 2005-2019. To understand how many of them are available only in the WHO registry and not in clinicaltrials.gov, we first removed the WHO trials having clinicaltrilas.gov as source, obtaining 18,234 trials.

After preprocessing (lowercase and punctuation removal) we compared the “Official” and “Brief” titles available in clinicaltrials.gov with the “Scientific” and “Official” titles available for WHO trials. For the comparison we used the optimal string alignment distance (function stringdistmatrix in R package stringdist) between titles and computed the percentage of change over the total length of the WHO title.

To calibrate the search algorithm and empirically establish a cutoff point able to best discriminate between overlapping and nonoverlapping trials, we manually checked 100 trials randomly extracted with stratification by % overlap bin, as follows: 25 with overlap 70-100%, 25 with overlap 40-70%, 25 with overlap 20-40% and 25 with overlap 0-20%. We then manually compared phase, size, interventional arms, primary/secondary endpoints and secondary ID to label each entry as overlapping or not, and computed a sensitivity-specificity ROC (Supplementary Figure S1). The optimal threshold was found at 76.6%, but we observed no differences in the True Positive rate when a threshold equal to 75% was selected (Supplementary Table S3). We thus chose 75%, obtaining specificity and sensitivity equal to 0.95.

We then set an additional constraint on the WHO title length: if the length is lower than 100, then the matching threshold was fixed at 90%. With this choice, specificity increased to 0.98 with sensitivity remaining high (0.95, Supplementary Table S3).

After applying those rules, we observed that 14,331 trials present in the WHO registry (WHO-specific, 31.4% of the total WHO trials) are not available in clinicaltrials.gov.

As in the WHO registry the geographic information is present but at national/regional scale rather than at the city level, we analysed the national distribution of WHO-specific trials. In the last ten years (2008-2019), the percentage of countries with more than 25% of WHO-specific trials was higher in the Upper-middle income class (among 10 and 20%, see Supplementary Figure S2A). In 2019, 19 countries had > 25% WHO-specific trials (Supplementary Figure S2B) and belong mainly to the Lower-middle income class (Morocco, Ghana, India, Pakistan, Nigeria, Indonesia) with some notable exceptions in higher and upper middle classes (China, Japan). For these nations, CTG is likely underestimating the number of available trials, making results on their accessibility less reliable. Unsurprisingly, Iran was systematically identified as the nation with the highest rate of WHO-specific trials.

**Comparison between distance-based and travel cost-based indices**

To construct our accessibility index, we used only publicly data available for a large number of countries. However, some of the parameters are only a generalization of what happens in the real world: for example, the Euclidean distance between locations does not describe in detail the actual “price” that patients may have to pay to reach the closest clinical trial. Travel time or money cost have been used to quantify this price, but only in specific or local context, making their use on a general scale unfeasible.

In order to understand whether distance may provide an adequate approximation of travel cost in the computation of accessibility index, we compared our index with four indices computed from travel costs available for Italy ^4^.

In ref ^4^, Italy was divided in 371 “zones” ﻿representing a homogeneous aggregation of municipalities and defined generalized costs of traveling between different zones using a transport model which ﻿includes the entire Italian long-distance supply: roads, coaches, long-distance rail services, air services, and ferries. Their traveling costs are thus based on distance, time and toll and depends on the vehicle used (car or local public transportation). Additionally, generalized costs were computed after considering different economical profiles of the potential user: business, economy and family, described by different ﻿values for travel time, private car availability and average stay in the place of destination. Costs are available for all possible departure-destination couples.

For our aim, we selected the economy and family profiles, since entering a clinical trial does not concern a business travel. Generalized travel costs considered were thus the following: 1) CgE_STR: generalized cost for traveling by car with economy profile; 2) CgF_STR: generalized cost for traveling by car with family profile; 3) CgE_TPL: generalized cost for traveling by public transportation with economy profile; 4) CgF_TPL: generalized cost for traveling by public transportation family profile.

Since trial locations and grid points for dAI may not be the same available for traveling cost, we first associated to each trial location and grid point the closest zone (in terms of Euclidean distance); and then considered the zones associated to the grid points as departures, the zones associated to trial locations as destinations.

Once obtained the generalized cost associated to each grid point-trial couple, we substituted the term $d_{iJ}^{-\beta}$ in our accessibility index dAI(i) with the generalized cost (e.g. $CgE\_STR_{ij}$) and computed the resulting value. We perform this operation for the four types of cost profile, obtaining four new local (and then national) accessibility indices: Index_CgE_STR, Index_CgF_STR, Index_CgE_TPL, Index_CgF_TPL.

We checked the local accessibility distribution of the indexes (Supplementary Figure S4A) and the Gini Index obtained in 2019 (Supplementary Figure S5), as well as their national trends from 2005 to 2019 (Supplementary Figure S4B). Additionally, we compared our distance-based index with the four travel-based indices by Pearson’s correlation and by linear regression, both at local level (Supplementary Figure S5) and at national level (comparison of time trends, Supplementary Figure S6). National time trends were highly correlated (all values >0.96), whereas among local correlations were still generally high (>0.72), with maximal correlation for public transport-based profiles (0.77-0.81) which may be considered the most prevalent profiles for the patient population that may be enrolled in clinical trials.

**Accessibility on simulated configurations**

We compared results obtained for each configuration when adding studies to different combinations of sites (Supplementary Figure S11). We remove redundant/symmetric combinations (such as case B for configuration “AB”, which is symmetric to case A) to enhance clarity in the results representation.

Overall accessibility improves when more research sites are considered: the maximum value of accessibility over all the configurations is obtained for “ABCDE” when all the sites are powered ($A=1.76)$. Similarly, within configurations, the maximum accessibility is always obtained when all the available sites are powered (those cases are represented with blue dots in Supplementary Figure S11, panel A). However, we noticed differences in the impact of sites location on accessibility: with two available research points we obtained higher accessibilities when considered either the central point (configuration “AE”) or the furthest one (configuration “AC”).

Investigating further the role of the central point E, we observed that whenever it belongs to the configuration, adding studies in this site alone allows to increase accessibility much more than other single sites (see panel B Supplementary Figure S11: configuration “AE” has accessibility equal to $0.87$ for 10 studies in A and equal to $1.2$ for 10 studies in E). In particular, in configuration “ADE” we noticed that adding five studies in E ($A=1.2$) is better than simultaneously adding five studies in single sites A and D ($A=0.9$), with the additional worth of saving resources.

Starting from configuration “ABE”, we also created configurations with different population distributions (Supplementary Figure S10, panels C, D, E and F) and compared the accessibility levels of those new configurations with that obtained in the baseline “ABE” (Supplementary Figure S12). The configurations for decreased population were joined since they obtained exactly the same accessibilities on all cases; the same happened for configurations with increased population: the position of grid points with higher/lower population did not influence accessibility: configurations for decreased (and increased) population obtained exactly the same accessibilities on all cases. Despite this may be due to the reduced dimensions of the grid, for our analysis they were joined.

Increasing population reduces the maximum accessibility that can be obtained: for configuration “ABE” the maximum obtained is $A=1.50$, while only $A=1.46$ for the configurations “ABE_PL” and “ABE_PR” (increase population in left upper and right bottom corners). Diminishing population has instead the opposite effect: maximum level of accessibility increased to $A=1.54$.

Again, we noticed that geographical location is more relevant than number of trials in our synthetic configurations. Indeed, the presence of point “E” among the powered sites is enough to boost the accessibility; while adding studies in a heterogeneous way has less effect. For instance, adding 10 studies in A and 5 in B (case “2AB” Supplementary Figure S12) has lower effect on accessibility than adding 5 studies both to A and E (case “AE”). Similarly, case “2AE” and “A2E” do not show improved accessibility with respect to case “AE”.

**Supplementary tables**

**Table S1:** Statistics on distance between patients and clinical sites in the years 2015-2019 across all studied countries. Mean, standard deviation, median and mode values (Km) are reported.

| Year | Mean (SD) | Median | Mode |
| --- | --- | --- | --- |
| 2015 | 729.97 (882.39) | 363.23 | 101.33 |
| 2016 | 685.34 (819.70) | 350.47 | 92.26 |
| 2017 | 704.85 (860.97) | 346.13 | 101.46 |
| 2018 | 668.38 (847.95) | 343.60 | 101.03 |
| 2019 | 719.73 (890.99) | 335.65 | 104.86 |

**Table S2**: Sources of trials available in the WHO registry, with correspondent country and with the number of cancer/tumor trials coming from each source in the period 2005-2019. A link to the national registry is available.

| National registry | Link | Country | N. trials |
| --- | --- | --- | --- |
| ANZCTR | http://www.anzctr.org.au/ | New Zealand | 1206 |
| ChiCTR | http://www.chictr.org.cn/abouten.aspx | China | 1899 |
| Clinicaltrials.gov | https://clinicaltrials.gov/ | All | 27447 |
| CRIS | https://cris.nih.go.kr/cris/info/introduce.do?search_lang=E&lang=E | Republic of Korea | 3355 |
| CTRI | http://ctri.icmr.org.in/ | India | 938 |
| EU Clinical Trial Register | https://www.clinicaltrialsregister.eu/ | European countries | 3850 |
| German Clinical Trials Register (DRKS) | https://www.drks.de/drks_web/ | Germany | 657 |
| IRCT | https://www.irct.ir/ | Iran | 810 |
| ISRCTN | https://www.isrctn.com/ | All (includes non-randomized studies) | 1067 |
| JPRN | 1. <https://www.umin.ac.jp/ctr/index.htm> 2. <https://jrct.niph.go.jp/> | Japan | 6416 |
| LBCTR | https://lbctr.moph.gov.lb/ | Lebanon | 3 |
| Netherlands Trial Register | https://www.trialregister.nl/ | The Netherlands | 508 |
| PACTR | http://www.pactr.org/ | African countries | 62 |
| REBEC | https://ensaiosclinicos.gov.br/ | Brazil | 154 |
| REPEC | https://ensayosclinicos-repec.ins.gob.pe/ | Peru | 192 |
| RPCEC | https://rpcec.sld.cu/ | Cuba | 54 |
| SLCTR | https://slctr.lk/ | Sri Lanka | 8 |
| TCTR | http://www.thaiclinicaltrials.org/ | Thailand | 75 |

**Table S3**: Comparison of different thresholds to match trials from WHO and clinicaltrials.gov. Metrics for the introduction of the additional rule (if the title length is lower than 100, then threshold is 90%) are also reported. TP=True Positive, FP=False Positive, TN=True Negative, FN=False Negative.

| Threshold | Accuracy | Sensitivity | Specificity | TP | FP | TN | FN |
| --- | --- | --- | --- | --- | --- | --- | --- |
| 76.6% | 0.95 | 0.95 | 0.95 | 76 | 4 | 19 | 1 |
| 75% | 0.95 | 0.95 | 0.95 | 76 | 4 | 19 | 1 |
| 75% + additional rule | 0.97 | 0.95 | 0.98 | 78 | 1 | 19 | 2 |

**Table S4:** Number of clinical trials for each agency/phase in 2019. Relative increment with respect to 2005 in parenthesis. No relative increment is considered for cases not observed in 2005

|  | **Early phase 1** | **Phase 1** | **Phase 1/2** | **Phase 2** | **Phase 2/3** | **Phase 3** | **Phase 4** | **N/A** | **TOTAL** | **Chi-squared pvalue** |
| --- | --- | --- | --- | --- | --- | --- | --- | --- | --- | --- |
| **Industry** | 23 | 376  (240%) | 169  (186%) | 174  (-38%) | 13  (44%) | 145  (6.6%) | 12  (-70%) | 39  (680%) | 951  (49%) | <2.2*10^-16^ |
| **Other** | 108  (1700%) | 287  (77%) | 188  (39%) | 945  (14%) | 45  (50%) | 179  (-41%) | 68  (112%) | 1161  (504%) | 2981  (76%) | <2.2*10^-16^ |
| **NIH** | 0 | 17  (-46%) | 15  (15%) | 38  (-54%) | 4 | 10  (25%) | 0 | 2  (0%) | 86  (-38%) | 1.98*10^-11^ |
| **U.S. Fed** | 0 | 0  (-100%) | 1 | 4  (0%) | 1  (-50%) | 0  (-100%) | 0  (-100%) | 2  (-82%) | 8  (-69%) | 0.39 |
| **TOTAL** | 131  (2083%) | 680  (120%) | 373  (80%) | 1161  (-3%) | 63  (54%) | 334  (-26%) | 80  (8%) | 1204  (473%) | 4026  (61.5%) |  |

**Table S5**: Summary of countries without any cancer clinical trial in the period 2005-2019 and those with at least one cancer clinical trial every year by income class

|  | No trials | Trials every year |
| --- | --- | --- |
| High income | Trinidad and Tobago | Austria, Belgium, Croatia, Czech Republic, Denmark, Estonia, Finland, France, Germany, Greece, Hungary, Ireland, Italy, Latvia, Lithuania, Netherlands, Norway, Poland, Portugal, Slovakia, Slovenia, Spain, Sweden, Switzerland, United Kingdom, Israel, Japan, Republic of Korea, Saudi Arabia, Taiwan, United States, Canada, Chile, Puerto Rico, Australia, New Zealand |
| Upper-middle income | Macedonia, Lybia, Gabon, Namibia, Equatorial Guinea, Turkmenistan, Suriname, Guyana, Fiji | Bosnia and Herzegovina, Bulgaria, Romania, Russian Federation, Serbia, South Africa, China, Lebanon, Malaysia, Thailand, Turkey, Argentina, Brazil, Colombia, Peru, Mexico |
| Lower-middle income | Moldova, Angola, Cote d’Ivoire, Republic of Congo, Djibouti, Leshoto, Mauritania, Cambodia, Bhutan, Uzbekistan, Kyrgyzstan, Lao PDR, Timor Leste, Bolivia, Nicaragua, Vanuatu, Papua Nuova Guinea, Solomon Islands | Ukraine, Philippines, Egypt and India |
| Low income | Burundi, Benin, Burkina Faso, Niger, Mali, Central African Republic, Democratic Republic of the Congo, Eritrea, The Gambia, South Sudan, Sierra Leone, Somalia, Chad, Mozambique, Guinea Bissau, Liberia, Guinea, Togo, Afghanistan, Syria, Tajikistan, Yemen | - |
| Not classified | Kosovo, Western Sahara, Swaziland, Somaliland, Palestine, Democratic Republic of Korea, Northern Cyprus, New Caledonia, Falkland Islands | - |

**Supplementary figures**

**Figure S1:** Area Under the Curve (AUC) for the string distance algorithm used to define as “matching” studies from WHO and clinicaltrials.gov. The point on the AUC defines the optimal threshold.

B

A

**Figure S2**: additional studies in the WHO registry over those in clinicaltrials.gov A) percentage of countries with more than 25% of additional clinical trials in the WHO registry. Percentages are given for different income classes, which are represented by different colors. B) Countries with at least 25% of additional studies in WHO in 2019. The barplot represents the percentage of studies in the WHO registry over those in clinicaltrials.gov. Colors describe the income class. Percentages larger than 1500% were set to 1500% in order to visualize also lower ones.


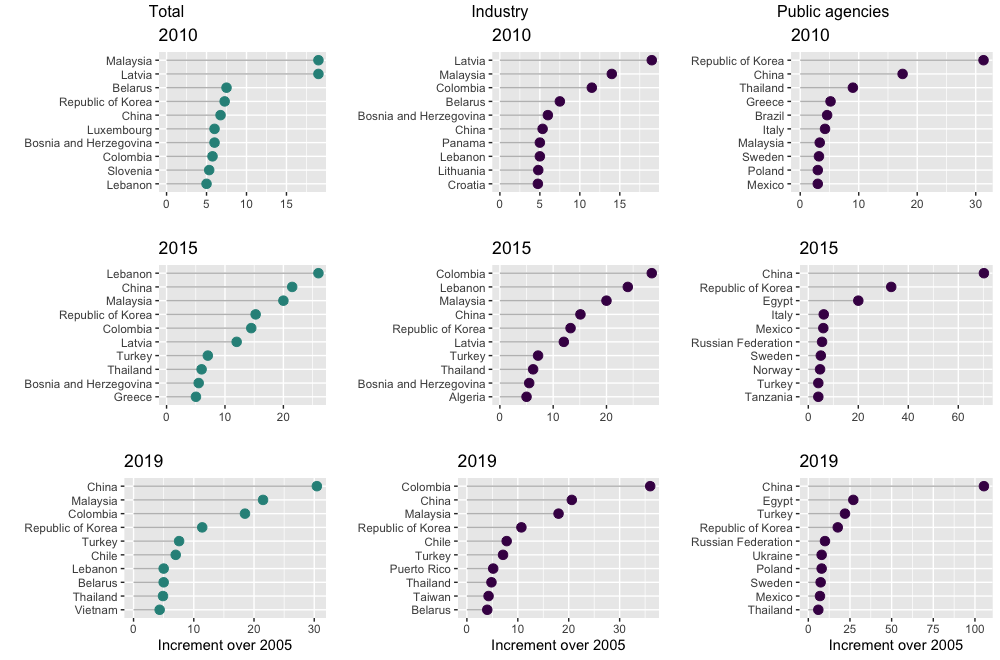


**Figure S3**: top 10 countries for total increase in studies (left panel), top 10 countries for increase of studies funded by industry (middle panel) and public agencies (right panel)

A

B

**Figure S4**: Comparison of Italian A) local accessibility in 2019 and b) trends of national accessibility in the period 2005-2019 as computed with our index based on distance between locations (index_2019 for panel A and NI for panel B) and four additional indices computed with travel costs by car (STR) or local public transportation (TPL) and for two economical profiles, “economy” (E) and “family” (F).

**Figure S5:** Linear regression of the four travel-cost based additional local accessibilities on our distance-based local accessibility in Italy in 2019. Additional indices are computed on costs by car (STR) or local public transportation (TPL) and for two economical profiles, “economy” (E) and “family” (F). Pearson’s correlation and Gini Index for 2019 are also displayed.

**Figure S6**: Linear regression of four travel-cost based additional national accessibilities (NI) trends from 2005 to 2019 on our distance-based national accessibility trends in Italy. Additional indices are computed on costs by car (STR) or local public transportation (TPL) and for two economical profiles, “economy” (E) and “family” (F). Pearson’s correlations among the national trends are also displayed.

**Figure S7:** Trends of national accessibilities in the period 2005-2019. The highlighted curve in green represents accessibility in the United States.


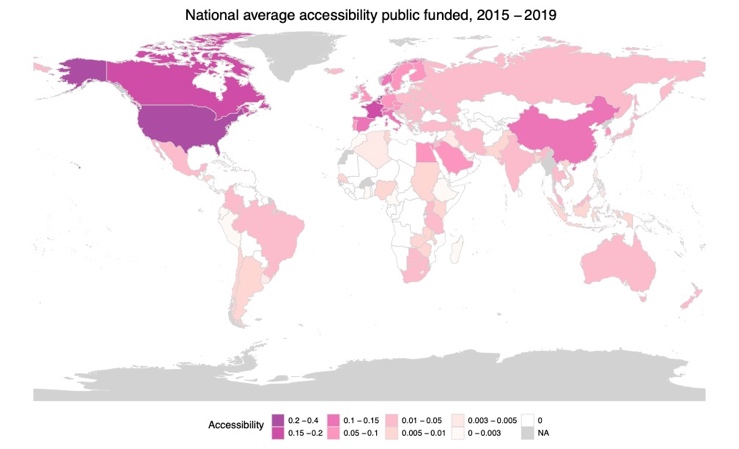

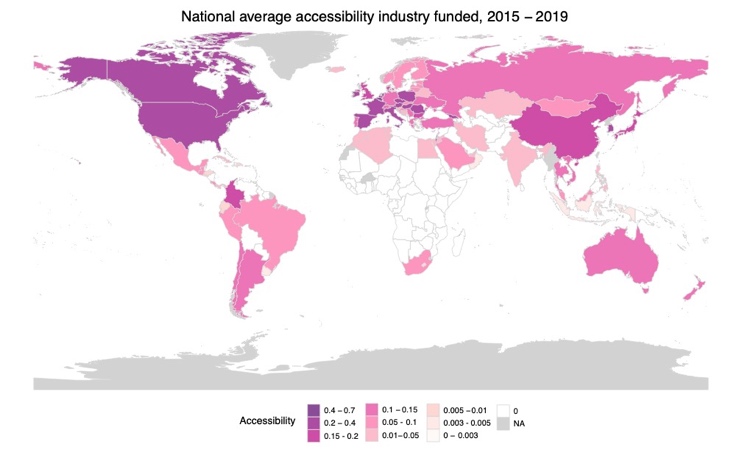


A

B

C


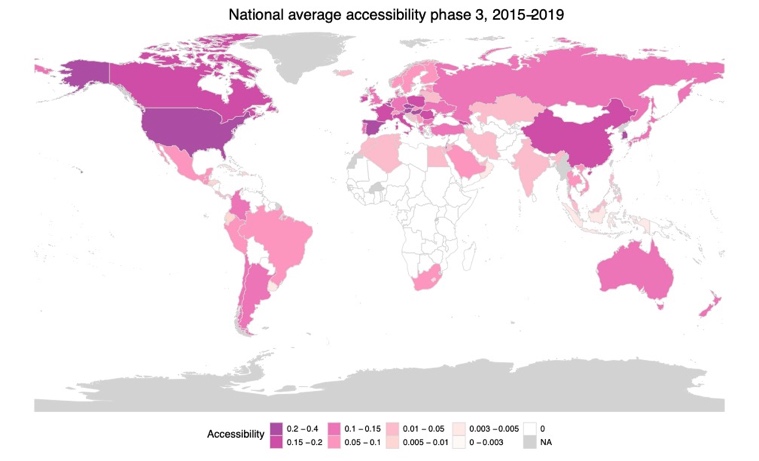


**Figure S8**: national average accessibility to A) public funded clinical trials, B) industry funded clinical trials and C) Phase 3 clinical trials in the period 2015-2019. Dark shadows of color are used for countries with higher national accessibility. White represents countries with no clinical trials in the years 2015-2019, grey is used for countries without available information.

**Figure S9:** Gini Index after removal of countries with more than 25% addition of WHO studies. Left panel: global Gini Index. Right panel: Average Gini index by income class. Income class are represented with different colors.


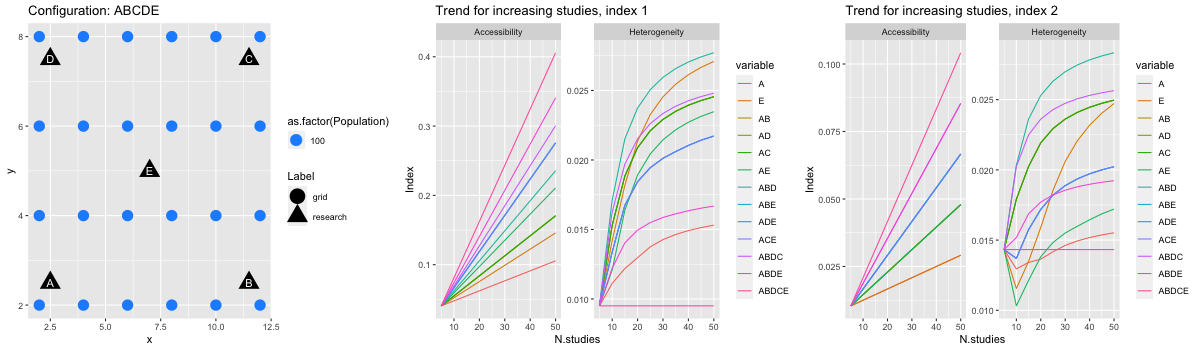

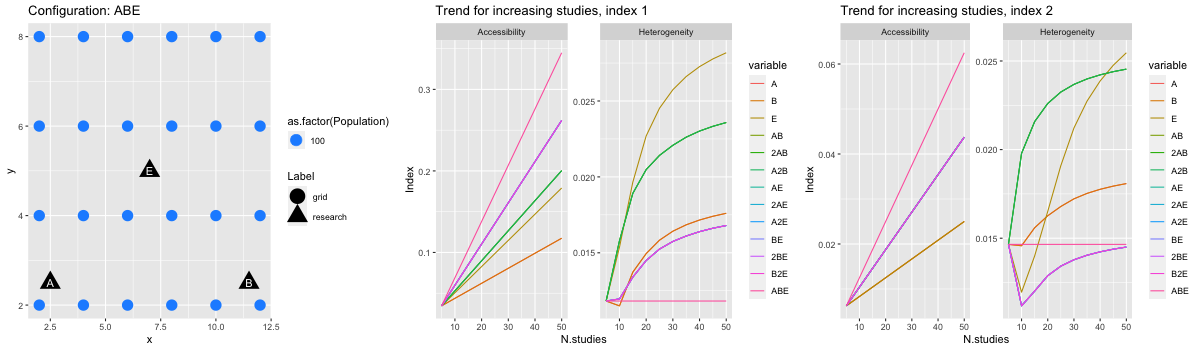

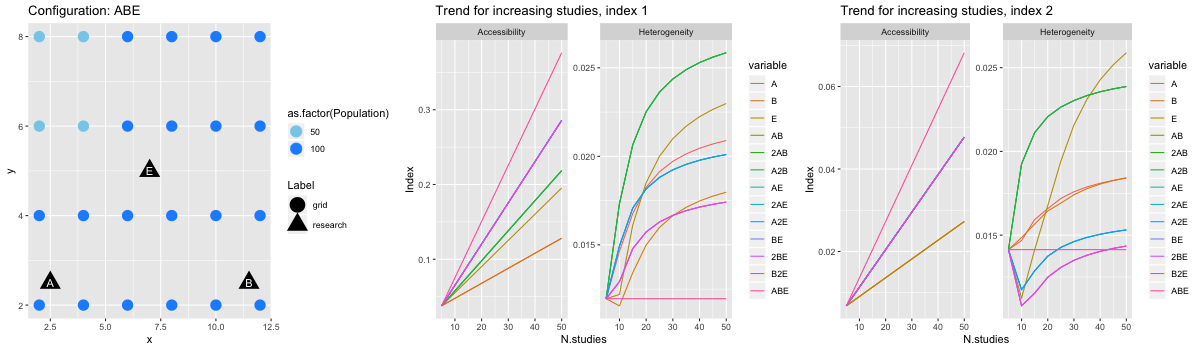

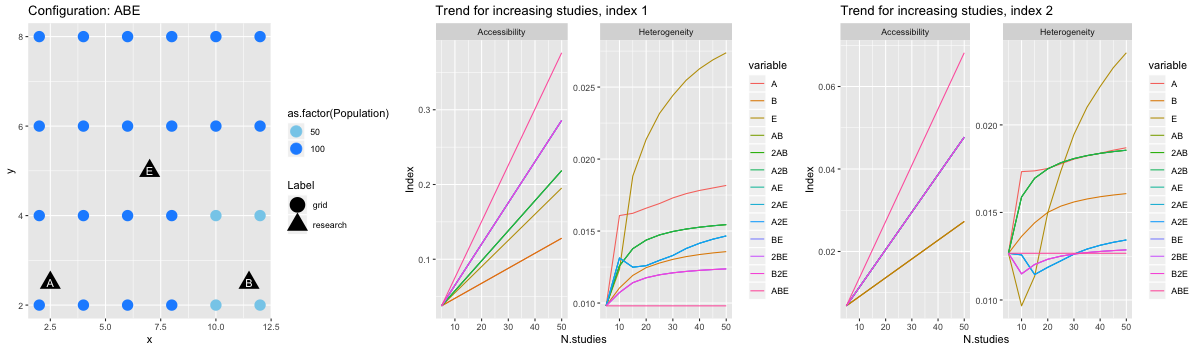

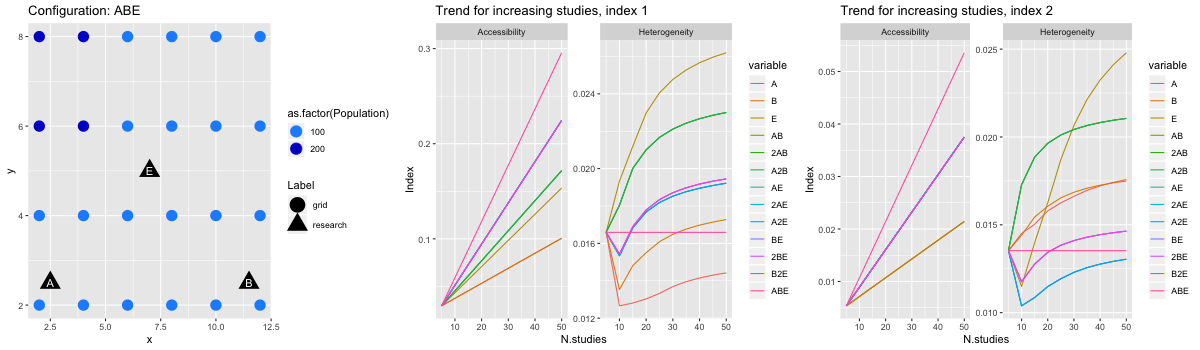

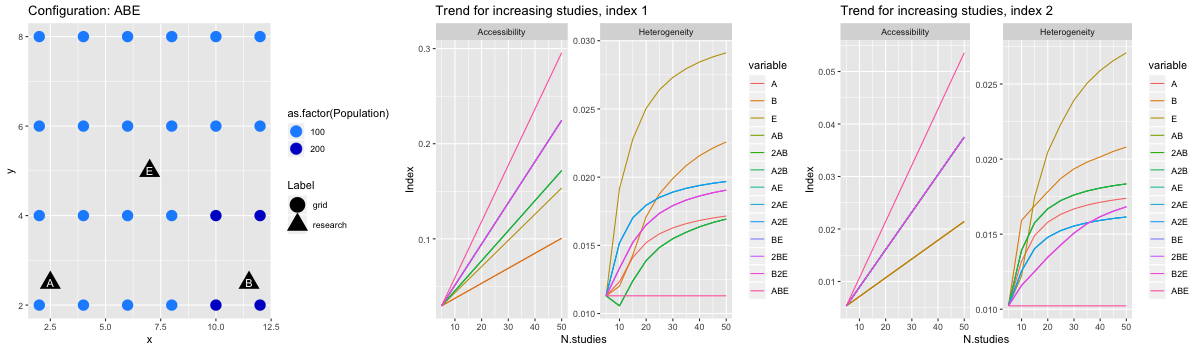


F

E

D

C

A

B

**Figure S10:** Examples of simulated configurations. Dots are grid points, with intensity color representing the population (50, 100 and 200), triangles represent “research points” (A, B, C, D, E)

A) Configuration “ABCDE” has all the “research points” and constant population=100

B) Configuration “ABE” has only research points A, B and E and constant population=100.

C) Configuration “ABE_LL” is created from configuration “ABE” with light blue dots in the left upper corner having less population=50

D) Configuration “ABE_LR” is configuration “ABE” with light blue dots in the right bottom corner having less population=50

E) Configuration “ABE_PL” is configuration “ABE” with dark blue dots in the left upper corner having higher population=200

F) Configuration “ABE_PR comes from configuration “ABE” with dark blue dots in the right bottom corner having higher population=200

A

B

**Figure S11**: Comparison of accessibility obtained for different configurations of research points. Configurations are on the x axis of both panels. Redundant/symmetric combinations are removed for clarity. A) Maximum accessibility obtained for each configuration. Blue dots represent the combination of sites with the maximum accessibility, with the correspondent label. Grey dots represent the other possible cases (e.g. for configuration “AE” the grey dots represent case A and case E, while blue dot is case “AE”); B) accessibility obtained for cases in configurations considering research site E. Cases are displayed as a triangle if they contain E, otherwise as a circle. The colors represent the number of sites per each case. For example, for configuration “AE”, the red circle is case A, the red triangle is case E, the blue triangle is case AE

**Figure S12**: Comparison of accessibility obtained on simulated configurations based on “ABE” with increased or decreased population. Cases on y axis represent sites with 5 additional studies. Dot shades correspond to configuration with base population (medium blue, configuration “ABE”), decreased population (light blue, configurations “ABE_LL” and “ABE_LR”) and increased population (dark blue, configurations “ABE_PL” and “ABE_PR”). The configurations for decreased (increased) population are joined since they obtained exactly the same accessibilities.

**References**

1. Tini G, Duso BA, Bellerba F, et al. Semantic and Geographical Analysis of COVID-19 Trials Reveals a Fragmented Clinical Research Landscape Likely to Impair Informativeness. *Front Med*. 2020;7(June):367. doi:10.3389/fmed.2020.00367

2. Lamont EB, Diamond SS, Katriel RG, et al. Trends in Oncology Clinical Trials Launched Before and During the COVID-19 Pandemic. *JAMA Netw open*. 2021;4(1):e2036353. doi:10.1001/jamanetworkopen.2020.36353

3. *Gridded Population of the World, Version 4 (GPWv4): Population Count, Revision 11*. Palisades, NY: NASA Socioeconomic Data and Applications Center (SEDAC); 2018. https://doi.org/10.7927/H4JW8BX5.

4. Beria P, Debernardi A, Ferrara E. Measuring the long-distance accessibility of Italian cities. *J Transp Geogr*. 2017;62(June 2016):66-79. doi:10.1016/j.jtrangeo.2017.05.006

5. Bray F, Ferlay J, Soerjomataram I, Siegel RL, Torre LA, Jemal A. Global cancer statistics 2018: GLOBOCAN estimates of incidence and mortality worldwide for 36 cancers in 185 countries. *CA Cancer J Clin*. 2018;68(6):394-424. doi:10.3322/caac.21492

6. OECD. *Handbook on Constructing Composite Indicators: Methodology and User Guide*.; 2008. doi:10.1111/jgs.13392

7. Shah SC, Kayamba V, Peek RM, Heimburger D. Cancer control in low- And middle-income countries: Is it time to consider screening? *J Glob Oncol*. 2019;2019(5):1-8. doi:10.1200/JGO.18.00200

8. Alessandretti L, Aslak U, Lehmann S. The scales of human mobility. *Nature*. 2020;587(7834):402-407. http://www.nature.com/articles/s41586-020-2909-1.
